# A tailored approach to building specialized surgical oncology capacity: Early experiences and outcomes in Malawi^☆^

**DOI:** 10.1016/j.gore.2018.10.001

**Published:** 2018-10-04

**Authors:** Lameck Chinula, Michael Hicks, Grace Chiudzu, Jennifer H. Tang, Satish Gopal, Tamiwe Tomoka, James Kachingwe, Leeya Pinder, Maya Hicks, Vikrant Sahasrabuddhe, Groesbeck Parham

**Affiliations:** a University of North Carolina at Chapel Hill, Department of Obstetrics & Gynecology, Chapel Hill, USA; b UNC Project-Malawi, Lilongwe, Malawi; c Malawi College of Medicine, Department of Obstetrics & Gynecology, Blantyre, Malawi; d Department of Obstetrics and Gynecology, Kamuzu Central Hospital, Lilongwe, Malawi; e Department of Obstetrics and Gynecology, University Teaching Hospital – Women and Newborn Hospital, Lusaka, Zambia; f University of North Carolina at Chapel Hill, Lineberger Comprehensive Cancer Center, Chapel Hill, USA; g National Cancer Institute, Bethesda, MD, USA; h Howard University College of Medicine, Washington, DC, USA

**Keywords:** Cervical cancer, Radical hysterectomy, Pelvic lymphadenectomy, Competency-based surgical training

## Abstract

**Objectives:**

Cervical cancer can often be cured by surgery alone, if diagnosed and treated early. However, of the cancer patients who live in the world's poorest countries less that 5% have access to safe, effective and timely cancer surgery. We designed a novel, competency-based curriculum to rapidly build surgical capacity for the treatment of cervical cancer. Here we report experiences and early outcomes of its implementation in Malawi.

**Methods:**

Curriculum implementation consisted of preoperative evaluation of patients and surgical video review, discussion of surgical instruments and suture material, deconstruction of the surgical procedure into critical subcomponents including trainees walking through the steps of the procedure with the master trainers, high-volume surgical repetition over a short time interval, intra-operative mentoring, post-operative case review, and mental narration. This was preceded by self-directed learning and followed by clinical mentorship through electronic communication and quarterly on-site visits.

**Results:**

Between June 2015–June 2017, 28 patients underwent radical abdominal hysterectomy with bilateral pelvic lymphadenectomy. The first 8 surgeries were performed over 5 days. After the 7th case the trainee could perform the procedure alone. During and between quarterly mentoring-visits the trainee independently performed the procedure on 20 additional patients. Major surgical complications were rare.

**Conclusions:**

Life-saving surgical treatment for cervical cancer is now available for the first time, as a routine clinical service, in Central/Northern, Malawi.

## Highlights

• Rapidly building surgical capacity for the treatment of cervical cancer in Malawi.
• Initiation of a sustainable gynecologic oncology service platform in Malawi.
• A practical public health solution to cancer control in a region of severe poverty.

Using a nontraditional approach to surgical training, we significantly shortened the time interval required to impart the technical skills required to perform a surgical procedure central to the treatment of invasive cervical cancer.

## 1 Introduction

Surgery is a cornerstone of cancer care. By 2030 21.6 million people across the world will be diagnosed with cancer, of which 17.3 million will need surgery, over half (10 million) of whom reside in low and middle-income countries (LMICs) where access to safe, effective and timely surgery is extremely limited. (Sullivan et al., 2015; Bray et al., 2012) Building and scaling specialized surgical oncology capacity in low income settings, alongside awareness and early detection service platforms, can have significant and immediate impact (WHO, n.d.). The challenge is to ensure that surgical training programs are contextually relevant, effective, and reproducible.

In high income countries (HICs) the predominant model of postgraduate surgical oncology education consists of multi-year specialty training within accredited programs, supported by experienced board-certified oncologic surgeons, readily available anesthetic services, highly advanced medical technology, intensive care units, ubiquitous blood banking, and modern laboratory infrastructure (Sullivan et al., 2015). On the other hand, in most LMICs the healthcare providers performing oncologic procedures are generalists (general surgeons, gynecologists, general practitioners, medical officers) without formal, certified subspecialty training, who provide cancer care out of necessity. Quite often the healthcare systems within which they attempt to deliver care are frail and their target populations are plagued by heavy burdens of extreme poverty, communicable diseases and advanced malignancies. An example is Malawi, a sub-Saharan African (SSA) nation of 18.6 million people that has one of the world's lowest levels of social and economic development (United Nations Development Programme, n.d.), smallest physician-patient ratios (World Health Organization: Global Health Observatory (GHO) Data, n.d.), highest HIV prevalence rates (World Health Organization, 2017), largest incident TB cases among people living with HIV (National Statistical Office (NSO) [Malawi] and ICF, 2017), and highest cervical cancer mortality rates (Ferlay et al., 2014).

The age standardized rate of cervical cancer in Malawi (76/100,0000) (Ferlay et al., 2014) is the highest in the world. To try and help remedy this situation the Malawi Ministry of Health implemented a “screen and treat” cervical cancer prevention program in 2004 using visual inspection with acetic acid (VIA) and cryotherapy (Msyamboza et al., 2016). Although program uptake has been less than optimal, there is evidence that service platforms of this nature, when scaled, can result in increased detection not only of preinvasive disease but also of invasive cancer, a significant percentage of which will be early stage (Parham et al., 2015). While it well known that preinvasive lesions of the cervix can be easily and successfully eradicated with local therapies (thermal ablation, cryosurgery, loop electrical excision, etc.), many invasive cancers can also be successfully treated, some with surgery alone, and deaths prevented, if detected early and managed properly (Parham et al., 2010). Alternatively, if treatment services are not available or otherwise inadequate, there is the risk of creating “cervical cancer death reservoirs”, i.e., large pools of women who are at risk of dying from diagnosed but untreated or inappropriately treated invasive cervical cancers.

In this report, we present our experiences of implementing a novel, competency-based pilot training program designed to rapidly build specialized surgical capacity for the treatment of cervical cancer in Malawi.

## 2 Materials and methods

### 2.1 Teaching model

**Fig. 1:**
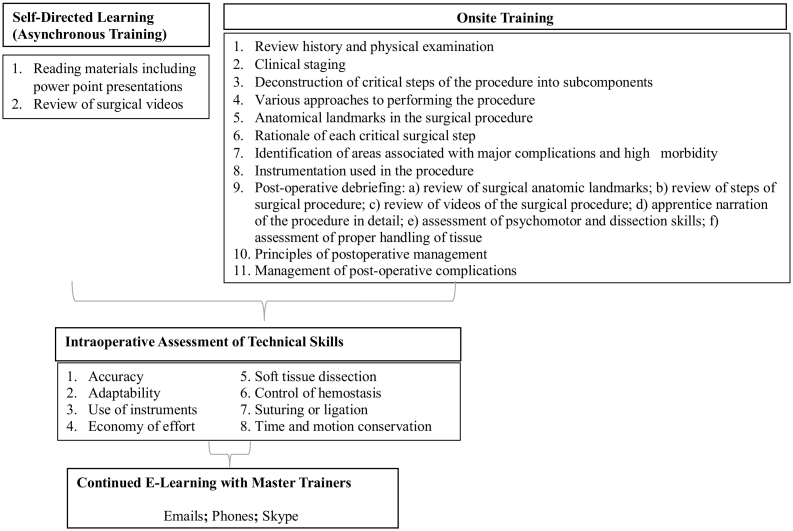
Overview of the competency-based surgical oncology training.

We implemented a modified version of surgical oncology training successfully piloted in Kenya.^12^ Our curriculum consisted of the following: (1) Preoperative evaluation of patients and video teaching sessions of the surgical procedures prior to each surgical case; (2) Hands-on, intraoperative mentoring, during which time the trainees were walked through the most critical subcomponents of the surgical procedure in a stepwise fashion (Fig. 1, Table 1) repeatedly, under the guidance of gynecologic oncology master trainers (U.S. board-certified gynecologic oncologists), with relevant discourse concerning the rationale behind each step; (3) Mental narration of the surgical procedure by the trainees several times a day, during which time they were instructed to review the rationale behind each step and to visualize potential intra-operative complications, specifically injury to major blood vessels, bowel, ureters and urinary bladder, corrective responses, and postoperative follow up. The importance of establishing a code of ethics in the operating room, awareness of all pertinent activities, and elimination of any potential distractions was stressed; (4) Post-surgical debriefing which focused on lessons learnt and difficulties encountered during the procedure; (5) Quarterly visits by the master trainer; (6) Distance mentoring in between visits using telecommunications.

**Table 1:** Subcomponents of the surgical procedure.

Mobilization of the ureters from the medial leaves of the broad ligament peritoneum and parametrium to their points of entry into the bladder, including identification and ligation of the uterine arteries at their origin from the internal iliac arteries
Division of the cardinal ligaments and parametrium
Mobilization of the rectum from the posterior cervix and vagina; identification and division of the uterosacral ligaments
Mobilization of the bladder from the anterior cervix and vagina; identification and division of the vesicouterine and vesicocervical ligaments

### 2.2 Training site

The training was implemented in the Department of Obstetrics and Gynecology at Kamuzu Central Hospital (KCH), a 1000-bed national teaching hospital in the capital city of Lilongwe, Malawi. For cancer care, KCH serves the entire Central and Northern region of Malawi. KCH also has a Cancer Clinic that operates daily and sees approximately 75–100 adult cancer patients each week. The Department of Obstetrics and Gynecology has 2 functioning surgical theaters: one is reserved for emergency surgeries, and the other is used for elective obstetrics and gynecologic cases. Both were used daily during the week of intensive training. There is also a weekly colposcopy and large loop electrosurgical excisional procedure (LEEP) clinic that attends to patients referred from VIA sites. In July 2011, KCH opened the first diagnostic pathology laboratory with collaborative efforts between the University of North Carolina Project-Malawi (UNCPM), University of North Carolina at Chapel Hill, and Malawi Ministry of Health. The laboratory is now a region leading resource, with immunohistochemistry (IHC) and telepathology services, (Gopal et al., 2014; Mabedi et al., 2014; Gopal et al., 2013a; Gopal et al., 2013b) and a turn-around time for histopathology results of 2 weeks. Prior to this, pathology services were only available in Blantyre (Malawi's second largest city), which is 360 km away from Lilongwe. All biopsies and surgical specimens were evaluated in this laboratory.

### 2.3 Personnel

The surgical trainees were Malawian board-certified obstetrician/gynecologists who expressed a keen interest in learning how to perform a radical abdominal hysterectomy and pelvic lymphadenectomy. The trainees were strongly supported by the leadership of KCH, as the training was seen as a step towards the eventual development of a gynecologic oncology service within its Department of Obstetrics and Gynecology. The two U.S. board-certified gynecologic oncology master trainers (MH, GP) have over 50 years of surgical experience between them, much of which has been spent practicing medicine and teaching in U.S. sites with high cervical cancer rates (Detroit, Michigan; South Central Los Angeles, California; Little Rock, Arkansas). Both were trained during a period when open laparotomy was the standard approach for cervical cancer surgery. Over the past 20 years, they have worked together in SSA and the West Indies leading cervical cancer screening and radical oncologic surgical demonstration seminars. One (GP) has lived and worked in Zambia since 2005, as a professor of gynecologic oncology at Women and Newborn Hospital - University Teaching Hospital of Zambia.

### 2.4 Pre-operative assessment and postoperative care

Patients with suspicious cervical lesions were initially identified in the KCH gynecology clinics, underwent cervical biopsy for histologic assessment, and clinically staged by the trainee using FIGO staging criteria. Those with stage IA2 – IIA1 disease were medically evaluated, cleared and then scheduled for surgery. General anesthesia was provided by certified anesthetic clinical officers; immediate recovery room monitoring by anesthetic clinical officers and nursing staff. Postoperative care was provided in the gynecologic ward of KCH.

We analyzed the 28 surgical cases that were accrued during the first 24 months of program implementation. Demographics, HIV status, clinical stage of disease, blood loss, blood transfusions, histopathologic results and surgical complications were documented.

## 3 Results

Between June 2015 and June 2017, 28 patients with early stage invasive cervical cancer underwent radical abdominal hysterectomy and bilateral pelvic lymphadenectomy (Table 2). Their median age was 46 years (IQR 40–51), median parity 5 (IQR 3–8), and of the 26 patients with known HIV status 38% (*n* = 10) were positive. The vast majority (75%, *n* = 21) were FIGO stage IB1. All had histopathological confirmation of invasive cervical cancer prior to surgery.

**Table 2:** Baseline characteristics of the surgical cases.

	Surgical cases, *N* = 28	Percentage
Age (years)		
<40	6	21.4
≥40	22	78.6
HIV status		
Negative	16	57.1
Positive	10	35.7
Unknown	2	7.1
Parity		
<5	9	32.1
≥5	19	67.9
Pre-operative cervical histopathology		
Squamous cell carcinoma	26	92.8
Adenocarcinoma	2	7.1
FIGO clinical stage		
IB1	21	75.0
IB2	2	7.1
IIA1	5	17.9
Pre-operative hemoglobin (g/dl)		
<11	2	7.1
≥11	20	71.4
Missing data	6	21.4

During the initial 5-day onsite practicum 8 patients underwent surgery. The first 7 cases were performed by the trainee with the assistance of a master trainer. On the final day the 8th case was performed exclusively by the trainee, unsupervised. The trainee continued to independently perform the procedure on 20 additional patients during the period of this report.

### 3.1 Surgical outcomes

The median post-surgery hospital stay was 8 days (IQR 4–8). Due to the fact that many patients reside in distant rural areas with little access to local health care, we try to ensure they are past the risk period for acute post-operative complications before discharge. Of the 28 patients, 19 had accurate data on blood loss. Sixty-three percent (*n* = 12) had an estimated blood loss of 1000 ml or more. Approximately 61% (*n* = 17) required blood transfusions (Table 3).

**Table 3:** Surgical outcomes of the cases.

	Surgical cases, N = 28	Percentage
Blood loss (ml)		
<1000	7	25.0
≥1000	12	42.8
Missing data	9	32.1
Blood transfusion		
Yes	17	60.7
No	5	21.4
Missing data	6	17.9
Histopathology of surgical specimens		
Cervix		
No residual disease	3	10.7
aCIN 3	2	7.1
Squamous cell carcinoma	22	78.6
Adenocarcinoma	1	3.6
Pelvic lymph nodes		
Negative	20	71.4
Positive	7	25.0
Not reported	1	3.6
Vaginal margins		
Negative	27	96.4
Not reported	1	3.6
Parametria		
Negative	27	96.4
Not reported	1	3.6
Post-operative complications		
Hemorrhage	1	3.6
Urologic injury	1	3.6
Infection/intestinal injury/Thromboembolism	0	0

a CIN: Cervical intraepithelial neoplasia.

### 3.2 Complications

Of the 28 patients who underwent surgery, one experienced severe hemorrhage (approximately 4000 ml) from inadvertent surgical interruption of the pre-sacral venous plexus during pelvic lymphadenectomy. The bleeding was successfully brought under control intra-operatively, however, the patient later succumbed to hypovolemic shock during the post-operative period due to lack of blood products for transfusion and the absence of intensive care capability. This major complication occurred during the first 6 months the trainee was performing surgery independently. The only other major complication was a vesicovaginal fistula which occurred 7 days after surgery but closed spontaneously with conservative management, i.e., transurethral urinary bladder drainage for 2 weeks (Table 3).

### 3.3 Histologic outcomes

Bilateral pelvic lymphadenectomies were successfully performed on all patients and the vast majority (71%, *n* = 20) were negative for involvement with cancer. Twenty-seven surgical specimens had negative parametrial and vaginal margins. The margin status was not reported on one case. Squamous cell carcinoma was the predominant histologic subtype (Table 3).

## 4 Discussion

Using a nontraditional approach to surgical training, we significantly shortened the time interval required to impart the technical skills required to perform a surgical procedure central to the treatment of invasive cervical cancer. Capable and motivated individuals from resource-limited settings that are trained in high-income western institutions often experience difficulties when they return to practice in their home environments (Magrath & Sutcliffe, 2014). Many invariably end up staying in the healthcare setting where they trained, resulting in a drain on capable manpower in LMICs. Training professionals in their home settings allows them to acquire skills in the environment with which they are familiar in terms of available resources and its accompanying challenges (Morhason-Bello et al., 2013). It also potentially gives them a personal stake in the success of their training and the development of cancer care infrastructure in their home institutions and countries. This can serve as a catalyst for building cadres of properly trained professionals in the future, who will then be able to train others.

While efforts to establish gynecologic oncology training programs in LMICS are underway, at present they are insufficient in number to meet the current and growing demand. In Africa, for instance, there are only 6 formal gynecologic oncology training programs (South Africa, Ethiopia, Ghana, Kenya, Uganda, and Zambia) (Johnston et al., 2017) and most are in their infancy. While necessary and commendable, the establishment of such programs requires considerable resources, infrastructure, commitment and time to develop and mature. A first or parallel step might consist of immediate onsite training of LMIC general surgeons and general gynecologists in a limited repertoire of cancer surgery procedures, tailored to treat a country's most common and surgically amenable cancers, as suggested by the Lancet Oncology Commission on Global Cancer Surgery following their recent investigation of global surgery disparities (Sullivan et al., 2015). In line with this recommendation we implemented a competency-based approach to targeted skills building in Lilongwe, Malawi that brought a general gynecology trainee to a level where a radical abdominal hysterectomy with bilateral pelvic lymphadenectomy could be competently performed in one (1) week, after repetitively performing 8 cases. Twenty (20) of the 28 women had negative pelvic lymph nodes and surgical margins, indicating that the surgical procedures were potentially curative for women who were formerly destined for death. Such a rapid method of achieving competency should not be considered inadequate, as many of the current U.S. gynecologic oncology fellows perform only 5–10 radical abdominal hysterectomies during their entire 2 years of clinical fellowship training. The Accreditation Council for Graduate Medical Education in the United States recently set the minimum number of cervical cancer treatment cases for gynecologic oncology fellows at 10 cases per fellow (inclusive of minimally invasive and open approaches, and radiation treatment with brachytherapy) over the course of their fellowship training (Accreditation Council for Graduate Medical Education (ACGME), 2018). Some of these cases are not performed by individual fellows but instead are shared among fellows, diluting the actual surgical experience. More importantly, their cases are usually not done repetitively, as was the case with our training program.

Effective major surgery of any type requires adequate ancillary support and infrastructure. Cancelation of radical abdominal hysterectomy cases due to inadequate nursing, anesthesia staff, or lack of medications is not uncommon at the training health facility in Malawi. It is paramount that investments are made in these areas to ensure sustained surgical services.

One of the common complications of radical hysterectomy with pelvic lymphadenectomy is blood loss (Levrant et al., 1992). One patient in our series succumbed to hypovolemic shock in the post-operative period because of the lack of availability of blood products. However, in our series we were able to transfuse 61% percent of the patients that required blood. This level of transfusion is a major accomplishment in a very low income setting in Africa, where blood products are notoriously scarce. Of note, because of the high HIV prevalence among its adult population, the majority of blood donors in Malawi are high school students. To accommodate students, blood donations are usually conducted in schools. As a consequence blood product availability is at its nadir during school holidays. To solve this problem we developed a system whereby patients booked for any major surgical gynecologic procedures are asked to mobilize potential blood donors among their families, friends, and relatives. We then exchange the blood that is donated on their behalf by family members for hospital-banked blood that is required for the surgical procedures. We also track down any unused blood donated for other patients who have already undergone surgical procedures and re-allocate it to cancer patients who cannot find any potential donors. This requires concerted time and effort from both the blood bank staff and clinicians.

Our competency-based, intensive surgical oncology training approach can complement ongoing international efforts to expand formal gynecologic oncology training opportunities for physicians practicing in LMICs (Johnston et al., 2017). However, lack of a certification system for those trained under this arrangement can potentially demotivate local physicians from engagement. Internationally-recognized training programs and organizations such as the College of Surgeons of East, Central, and Southern Africa (COSECSA), the recently inaugurated Hematology and Oncology Society of Africa (HOSA), and International Gynecologic Cancer Society's (IGCS) Gynecologic Oncology Training Program, could potentially assist in the creation of standards of care, general curricula, and international examiners for trainees in surgical intensification programs such as ours, across many African nations, for purposes of certification (Magrath & Sutcliffe, 2014; http://hosafr.org/index.php/hosa/constitution, n.d.; http://www.theg4alliance.org/about-college-of-surgeons-east-central-and-southern-africa, n.d.).

A major limitation to the expansion of our training model to other sites across Africa is the lack of availability of master trainers who can work in limited resource environments and have experience in open surgical approaches. The absence of economic incentives, long and sometimes unpredictable travel times, language and cultural barriers, all work to limit their availability. Gynecologic oncologists currently undergoing training in high income countries have limited exposure to open approaches to radical abdominal hysterectomies, due to the shift from open to minimally-invasive surgical procedures, i.e., laparoscopic or robotic radical hysterectomies using high tech platforms that have very little if any presence and are difficult to sustain in SSA. There is a need for an organizational vehicle that facilitates partnerships between master surgeons experienced with open approaches to surgery and LMIC surgical trainees, to help build surgical oncology capacity in resource-limited settings where open surgery remains the standard surgical approach.

Although our training intervention was highly successful in the transfer of surgical skills after the first 8 cases (technical performance of the procedure, selection of surgical instruments, choice of suture material, etc.), optimal surgical proficiency (efficient hand and surgical tool motion, surgical dexterity, etc.) was not generally achieved until after approximately 20 total surgical procedures had been performed, over an 8-month period. This observation of needing more time to gain surgical adroitness is no different than what is seen with newly trained gynecologic oncology fellows in the U.S. after 2 years of clinical fellowship training. It was collectively concluded that more intensive pre-training and more frequent mentoring could have led to the acquisition of surgical proficiency in less time, with fewer procedures. Recent advances in web-based video monitoring systems may help facilitate this process (Budrionis et al., 2014).

This report has the methodological weakness of being a descriptive retrospective review of our surgical outcomes. Some data was missing on pre-operative hemoglobin (6 patients), blood loss (9 patients), blood transfusions (6 patients), pelvic lymph node status (1 patient), vaginal margin (1 patient) and parametrical involvement (1 patient). This was due to loose papers being lost when files were in storage because we had no digital electronic medical record system in our facility. The addition of a prospective electronic database system would allow us to overcome this problem in the future. However, it should be noted that although the above missing items are important, the stratification and description of these categories were not the intent of this project. We were still able to observe and document the most important metric, histopathology of the surgical specimens, to determine the success of the program. Discussing the number of pelvic lymph nodes removed and proposed adjunctive treatment for patients with positive lymph nodes or surgical margins, are beyond the scope of this report. Ultimately this will depend on the resources available in each local setting. While radiation therapy was not available in Malawi during the time of the program, as is the case in the majority of countries in SSA, the first radiation therapy center is due to open in 2019. These health system gaps only highlight the importance of early detection and the ability to perform proper surgery.

Global cancer surgery training in LMICs can be complex, and for a myriad of reasons. Some are system-related, others patient-related, and yet others related to the skill levels of trainees and their attitudes towards change (Magrath & Sutcliffe, 2014; Masamba, 2015). There are no one-size-fits-all solutions, and to be successful one must understand the context in which he or she is working and adjust, but not submit, to the circumstances. Our training curriculum significantly reduced the time required for mastery of the skills required to perform a single but very significant surgical oncologic procedure – radical abdominal hysterectomy, bilateral pelvic lymphadenectomy – critical for the treatment of invasive cervical cancer, the number one cause of cancer-related death in Malawi. It also resulted in the immediate establishment of a new surgical service, for a large population of at-risk women, that saves lives and is sustainable within the low resource context in which it was implemented.

## CDC disclaimer

The findings and conclusions in this report are those of the authors and do not necessarily represent the funding agencies.

## Conflicts of interest

The authors have no conflicts of interest to declare.
